# Influenza vaccination and major cardiovascular risk: a systematic review and meta-analysis of clinical trials studies

**DOI:** 10.1038/s41598-023-47690-9

**Published:** 2023-11-19

**Authors:** Fatemeh Omidi, Moein Zangiabadian, Amir Hashem Shahidi Bonjar, Mohammad Javad Nasiri, Tala Sarmastzadeh

**Affiliations:** 1 Department of Cardiology, Imam Hossein Hospital, Shahid Beheshti University of Medical Sciences, Tehran, Iran; 2 School of Medicine, Shahid Beheshti University of Medical Sciences, Tehran, Iran; 3 Endocrinology and Metabolism Research Center, Institute of Basic and Clinical Physiology Sciences, Kerman University of Medical Sciences, Kerman, Iran; 4 Clinician Scientist of Dental Materials and Restorative Dentistry, School of Dentistry, Shahid Beheshti University of Medical Sciences, Tehran, Iran

**Keywords:** Cardiology, Diseases, Health care, Medical research

## Abstract

Cardiovascular events remain a substantial global health concern, necessitating innovative strategies for prevention. This study aims to assess the potential impact of influenza vaccination on major cardiovascular events. A search of the medical English literature was conducted using PubMed/MEDLINE, EMBASE, and the Cochrane CENTRAL up to 1 August 2023. Meta-analysis and stratified analyses were performed to investigate specific outcomes, including myocardial infarction (MI), cardiovascular death, and stroke. Pooled relative risks (RR) along with their 95% confidence intervals (CI) were calculated to evaluate the associations. A comprehensive analysis was conducted on a total of 9059 patients, with 4529 patients receiving the influenza vaccine and 4530 patients receiving a placebo. Among patients who received the influenza vaccine, a notable reduction in the occurrence of major cardiovascular events was observed, with 517 cases compared to 621 cases in the placebo group (RR 0.70; 95% CI 0.55–0.91). The stratified analysis revealed a decreased risk of MI in vaccinated patients (RR 0.74; 95% CI 0.56–0.97) and a significant reduction in cardiovascular death events (RR 0.67; 95% CI 0.45–0.98). This study provides compelling evidence that influenza vaccination is associated with a decreased risk of major cardiovascular events, particularly myocardial infarction, and cardiovascular death. These findings highlight the potential of influenza vaccination as an adjunctive strategy in cardiovascular disease prevention. Further research and exploration of underlying mechanisms are warranted to elucidate the observed beneficial effects.

## Introduction

The significance of influenza vaccination has long been acknowledged in preventing seasonal flu infections, particularly in high-risk populations^1–3^. There are strong recommendations for influenza vaccination where six months and older people especially adults should be vaccinated against influenza^4^.

Cardiovascular diseases (CVDs) including hypertensive heart disease, ischemic heart disease, atrial fibrillation cerebrovascular disease like stroke, endocarditis, peripheral vascular disease, and other related cardiovascular diseases are a leading cause of morbidity and mortality worldwide^5,6^.

Differing viewpoints exist regarding the impact of influenza vaccination on CVDs. While certain observational investigations suggest a favorable correlation between influenza vaccination and the reduction in occurrences of cardiovascular incidents like acute myocardial infarction (MI), contrasting epidemiological studies propose the limited efficacy of influenza vaccines^7–10^.

Consequently, a comprehensive and updated study becomes imperative. This updated meta-analysis seeks to assess whether a connection exists between influenza vaccination and a decreased likelihood of experiencing cardiovascular events.

## Methods

### Search strategy

A search of the medical English literature was conducted using PubMed/MEDLINE, EMBASE, and the Cochrane CENTRAL up to 1 August 2023. A thorough review was conducted on randomized controlled trials (RCTs) exploring the potential link between influenza vaccination and the subsequent risk of developing CVDs.

The search terms were: myocardial disease and influenza vaccines (Table S1). This study adhered to the Preferred Reporting Items for Systematic Reviews and Meta-Analyses statement (PRISMA) for its design and reporting (Table S2) (Prospero ID: CRD42023450694)^11^.

### Study selection

The records obtained from the database searches were amalgamated, and redundancy was addressed through employment of EndNote X7 (Thomson Reuters, Toronto, ON, Canada). Subsequently, two evaluators (MZ and MJN) carried out individual assessments of the records, considering both their title/abstract and full text, in order to exclude any entries that did not align with the study's objectives.

The studies included in the analysis met the following criteria based on the PICOs:

*Participants:* The patients with a diagnosis of CVDs.

*Intervention:* Patients with CVDs who received influenza vaccine.

*Comparison:* Patients with CVDs who received a placebo.

*Outcome:* Lower risk of cardiovascular events.

Conference abstracts, case reports, and studies comparing high and low doses of influenza vaccination were excluded.

### Data extraction

Data extraction was conducted by two investigators (MZ and MJN) and recorded in a Microsoft Excel spreadsheet (XP professional edition; Microsoft Corp, Redmond, WA). The extracted information encompassed various key elements, including the first author's name, publication date, study type, definitions of cases and controls, the number of cases and controls, and the outcomes of the studies.

### Quality assessment

The quality assessment of the included studies was conducted by two reviewers (MJN and MZ) using the Cochrane tool^12^. In case of any discrepancies, a third reviewer (FO) was involved. This instrument encompasses a range of domains, which encompass random sequence generation, allocation concealment, blinding of both participants and personnel, blinding of assessors for outcomes, completeness of outcome data, and additional considerations such as selective reporting and potential sources of bias. Each study was categorized as having a low risk of bias when no concerns regarding bias were identified, a high risk of bias when there were concerns about bias or an unclear risk of bias when there was insufficient information available.

### Statistical analysis

The combined outcomes were presented as risk ratios along with corresponding 95% confidence intervals (CIs). To evaluate the level of variability among the studies, both the I2 value and *p*-value were employed. In cases where minimal statistical heterogeneity was observed (I2 ≤ 50% or *p* ≥ 0.1), the fixed-effect model was applied. Conversely, if substantial inter-study heterogeneity was identified (I2 > 50% or *p* < 0.1), the random-effects model was utilized. Assessment of between-study heterogeneity involved the application of Cochran's Q test and the I2 statistic. To assess potential publication bias, Begg's test was employed, where a significance level of *p* < 0.05 indicated noteworthy publication bias. All analytical procedures were conducted using Comprehensive Meta-Analysis Software, Version 3.0 (Biostat, Englewood, NJ).

## Results

**Figure 1:**
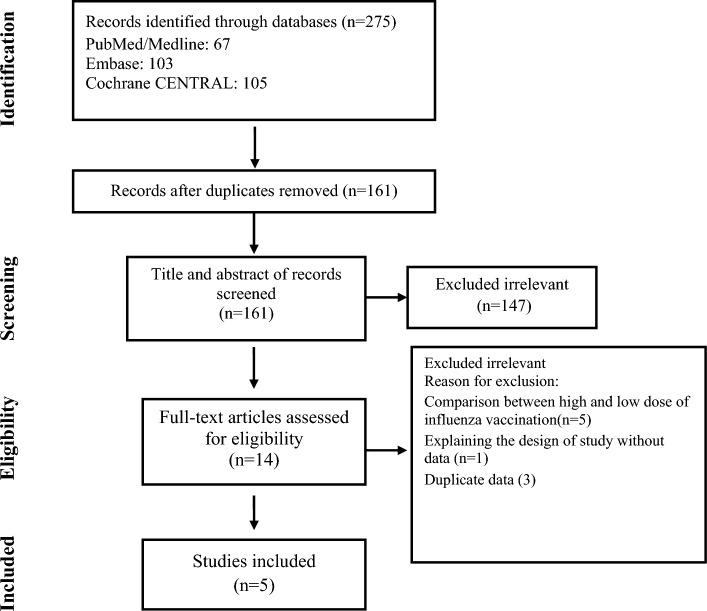
Flow chart of study selection for inclusion in the systematic review and meta-analysis.

Out of the 275 articles initially identified, five studies comprising 9059 patients met the predetermined inclusion criteria, as illustrated in Fig. 1^8,13–16^. The key characteristics of these selected studies are summarized in Table 1. Among the participants, a total of 4529 individuals were randomly assigned to receive a standard intramuscular influenza vaccination, while 4530 were assigned to receive an intramuscular placebo. The average age of the participants was 61.3 years, and they were followed up for an average duration of 9 months. The majority of the trials exhibited robust practices in terms of randomization, allocation concealment, and masking, aligning with the Cochrane criteria for high quality (low risk of bias), except for one trial (Table 2).

**Table 1:** Characteristic of included studies.

First author	Published year	Country	Mean Age	Definition of case (Number of participants)	Definition of control (Number of participants)	Outcome
Loeb^13^	2022	Multi-center	57	Vaccinated patients with heart failure and NYHA functional class II, III, and IV (2560)	Unvaccinated patients with heart failure and NYHA functional class II, III, and IV (2569)	IHD, Stroke, Cardiovascular death
Frobert^14^	2021	Multi-center	60	Vaccinated patients with MI (1272)	Unvaccinated Participants with MI (1260)	IHD, Stroke, Cardiovascular death
Phrommintikul^8^	2011	Thailand	66	Vaccinated patients with the acute coronary syndrome (n = 221)	Unvaccinated patients with the acute coronary syndrome (n = 218)	IHD, Stroke, Cardiovascular death
Ciszewski^15^	2008	Poland	60	Vaccinated patients with coronary artery disease (n = 325)	Unvaccinated patients with coronary artery disease (n = 333)	IHD, Cardiovascular death
Gurfinkel^16^	2002	Argentina	65	Vaccinated patients with MI or planned stenting (n = 151)	Unvaccinated patients with MI or planned stenting (n = 150)	IHD, Cardiovascular death

**Table 2:** Quality assessment.

Author	Random sequence generation	Allocation concealment	Blinding of participants and personnel	Blinding of outcome assessment	Incomplete outcome data
Loeb	Low	Low	Low	Low	Low
Frobert	Low	Low	Low	Low	Low
Phrommintikul	Low	Low	Unclear	Unclear	Low
Ciszewski	Low	Low	Low	Unclear	Low
Gurfinkel	Low	High	Unclear	Unclear	Low

### Pooled major cardiovascular events

**Figure 2:**
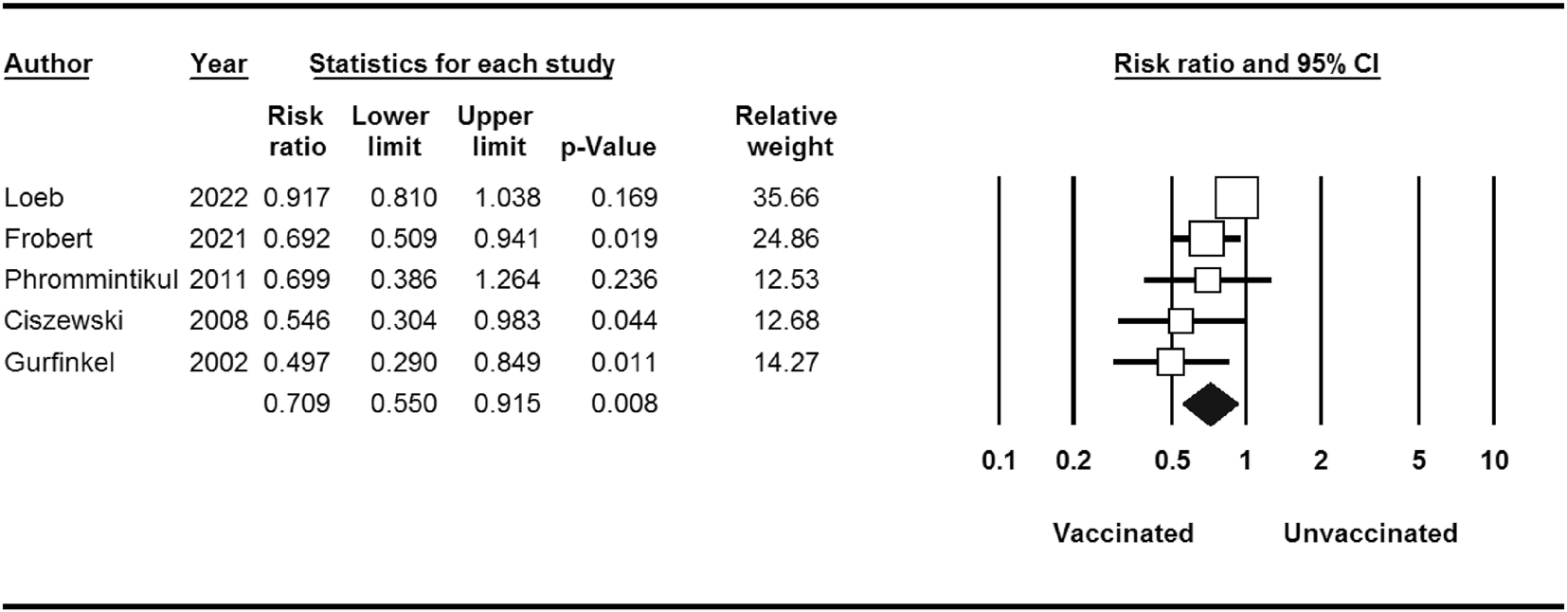
Major cardiovascular events for influenza vaccine versus control.

Within the cohort of 4529 patients who underwent influenza vaccination, a total of 517 individuals experienced significant cardiovascular events, in contrast to 621 cases among the 4530 patients who were administered a placebo. The calculated risk ratio (RR) was 0.70, indicating a noteworthy reduction in risk, accompanied by a 95% confidence interval (CI) of 0.55–0.91 (as depicted in Fig. 2).

### Pooled stratified analysis

**Figure 3:**
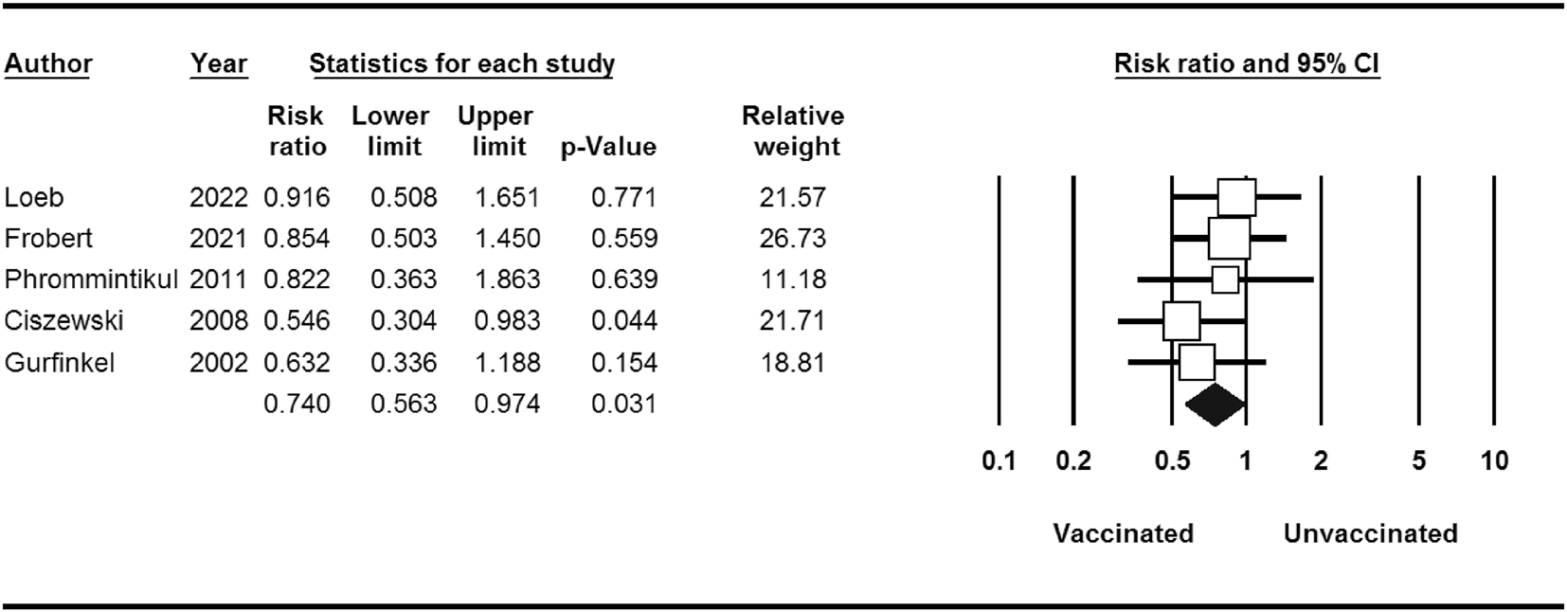
MI events for influenza vaccine versus control.

**Figure 4:**
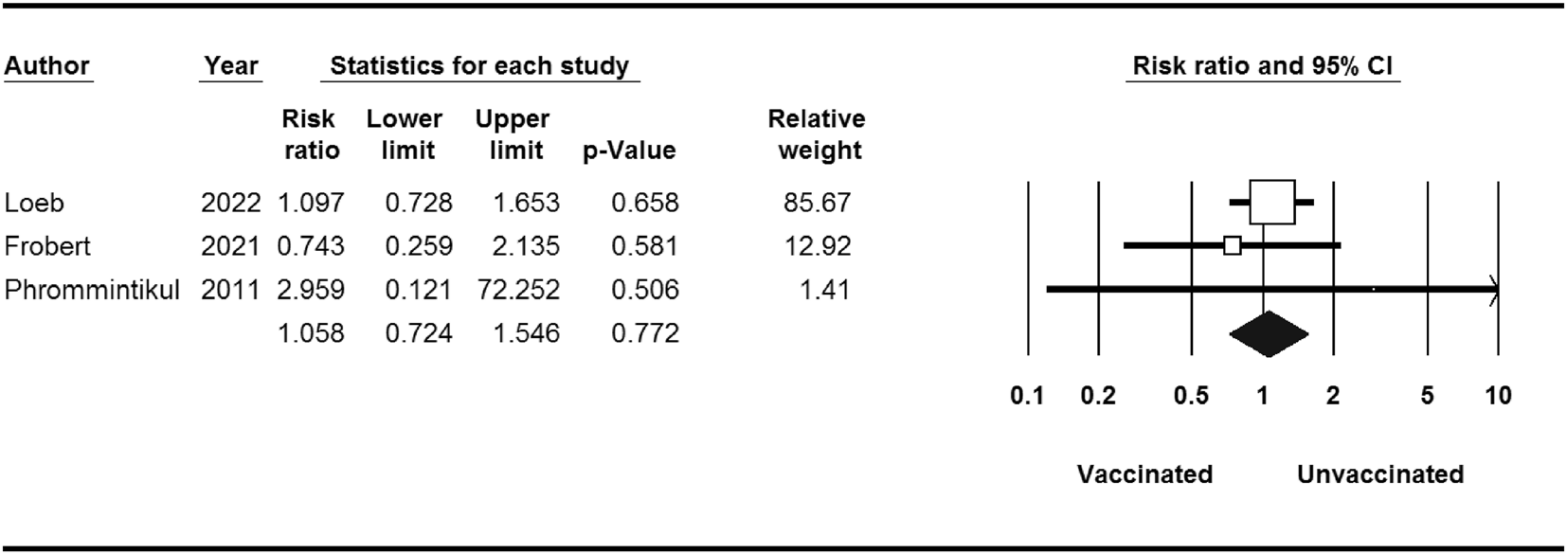
Stroke events for influenza vaccine versus control.

**Figure 5:**
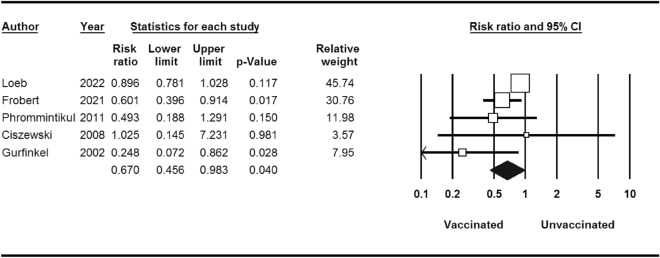
Cardiovascular death events for influenza vaccine versus control.

Stratified analyses are shown in Table 3. MI occurred in 86 of the 4529 patients who received influenza vaccine compared with 116 of the 4530 patients who received placebo (RR 0.74; 95% CI 0.56–0.97) (Fig. 3). The RR of developing cardiovascular death events after influenza vaccination was 0.67 (95% CI 0.45–0.98) (Fig. 4). Thus, influenza vaccination significantly decreased the risk of cardiovascular death (*P*-value = 0.04). There was not any significant association between influenza vaccination and risk of stroke (*P*-value = 0.7) (Fig. 5).

**Table 3:** Stratified pooled analysis.

Outcome	No. of study	No. of patients	Risk ratio (95% CI)/(*p*-value)	Heterogeneity I^2^ (%)/*p*-value	Begg test *p*-value
Major cardiovascular events	5	9059	0.70 (0.55–0.91)/0.00	58.16/0.04	0.80
Stratified analysis					
Myocardial infarction	5	9059	0.74 (0.56–0.97)/0.03	0.00/0.71	1.00
Stroke	3	8100	1.05 (0.72–1.54)/0.77	0.00/0.65	1.00
Cardiovascular death	5	9059	0.67 (0.45–0.98)/0.04	51.19/0.08	0.80

## Discussion

Revealing a compelling insight into the potential benefits of influenza vaccination, our comprehensive meta-analysis, based on the latest randomized controlled trial (RCT) data, demonstrates a significant interaction between influenza vaccination and the reduction of major cardiovascular events. Notably, patients who received the influenza vaccine experienced a remarkable risk reduction of over 20% in cardiovascular death. These findings underscore the potential impact of influenza vaccination in safeguarding against adverse cardiovascular outcomes among vulnerable patient populations.

### Mechanisms of the potential relationship

Understanding the potential mechanisms underlying this association is paramount to comprehending the clinical and public health implications of our findings^17^.

*Inflammation Modulation* Influenza infections are known to trigger systemic inflammation, and this inflammatory response may contribute to the progression of atherosclerosis and CVDs^18–20^.

*Immune System Modulation* Influenza vaccination activates the immune system, potentially influencing not only the response to influenza viruses but also the broader immune environment. A well-regulated immune system is essential for cardiovascular health, and the modulation of immune responses by vaccination may contribute to the observed cardiovascular benefits^18,19,21^.

*Prevention of Secondary Infections* Influenza infections can weaken the immune system, making individuals more susceptible to secondary infections^22^. Secondary infections, especially respiratory infections, can exacerbate cardiovascular conditions. Influenza vaccination's role in preventing these secondary infections may indirectly contribute to the reduction in CVDs.

*Stability of Atherosclerotic Plaques* Influenza infections have been implicated in the destabilization of atherosclerotic plaques, leading to acute cardiovascular events^23,24^. Influenza vaccination may play a role in maintaining the stability of these plaques, reducing the likelihood of rupture and subsequent cardiovascular complications.

### Comparisons with other studies

Our results align with the Behrouzi et al. meta-analysis published in 2022, which also demonstrated a decreased risk of CVDs in patients receiving influenza vaccination^25^. However, a more detailed analysis of the similarities and differences between our study and Behrouzi et al. is warranted.

In our study, we conducted stratified analyses based on MI, stroke, and cardiovascular death. This approach allowed us to focus specifically on a population with pre-existing CVDs, aiming to isolate and better understand the direct impact of influenza vaccination on individuals with established cardiovascular conditions. This differs from Behrouzi et al. as we deliberately excluded studies involving sample populations with specific comorbidities to ensure a more targeted examination. To delve deeper into these distinctions, it's crucial to note that our study's exclusion criteria were designed to minimize the influence of confounding factors. This deliberate choice to focus exclusively on a target population with pre-existing CVDs enhances the internal validity of our findings and provides a clearer picture of the direct relationship between influenza vaccination and cardiovascular outcomes. While our calculated RR for major cardiovascular events (0.70; 95% CI 0.55–0.91) was slightly higher than Behrouzi et al. (0.66; 95% CI 0.53–0.83), the consistency in direction and statistical significance reinforces the robustness of the observed association. This nuanced comparison underscores the importance of considering study design and inclusion criteria when interpreting the results of meta-analyses in the context of influenza vaccination and cardiovascular outcomes.

### Implications of the findings

The observed interaction between influenza vaccination and reduced cardiovascular mortality among patients with recent CVDs is both clinically and epidemiologically significant. Cardiovascular diseases, including heart attacks and strokes, remain the leading causes of mortality worldwide. The potential of influenza vaccination to yield substantial reductions in cardiovascular mortality in this vulnerable patient group warrants serious attention from healthcare providers, policymakers, and researchers.

### Clinical relevance and public health impact

By targeting patients with recent CVDs for influenza vaccination, healthcare providers have a potential opportunity to mitigate the risk of cardiovascular death in a cost-effective and widely available manner. Influenza vaccination programs could be tailored to prioritize this high-risk group, thus potentially reducing the overall burden on healthcare systems and improving patient outcomes.

## Conclusion

The evidence from the most recent meta-analysis of RCT data is compelling, revealing a significant interaction between influenza vaccination and the reduction of major cardiovascular events among patients with recent CVDs. This finding underscores the potential benefits of targeting this high-risk group for vaccination. Further research is warranted to elucidate the precise mechanisms driving this association and to explore the long-term impact of influenza vaccination on cardiovascular outcomes. In the meantime, healthcare providers and policymakers should take heed of these findings and consider prioritizing influenza vaccination for patients with recent CVDs as a feasible and potentially life-saving preventive measure.

### Supplementary Information

Supplementary Table S1.

Supplementary Table S2.

## Data Availability

The data used to support the findings of this study are included within the supplementary files.
