# Supplementary material for: Influenza vaccination and major cardiovascular risk: a systematic review and meta-analysis of clinical trials studies
Source: Sci Rep. 2023 Nov 19;13:20235. doi: 10.1038/s41598-023-47690-9 (PMC10658159; doi:10.1038/s41598-023-47690-9)
Supplement: Supplementary file 1 — Supplementary Table S1. [file 41598_2023_47690_MOESM1_ESM.docx]

Table S1. Search strategy for PubMed/Medline.

| **Search** | **Query** |
| --- | --- |
| #1 | (Myocardial Infarction[MeSH Terms]) OR Myocardial Infarction[Title/Abstract] OR Myocardial infarction [Title/Abstract] OR Cardiovascular[Title/Abstract] OR Atherosclerosis[Title/Abstract] OR Atrial fibrillation[Title/Abstract] OR Stroke[Title/Abstract] |
| #2 | (Influenza Vaccines[MeSH Terms]) OR Influenza Vaccine[Title/Abstract] |
| #3 | #1 AND #2 |
